# New and Upcoming Topical Treatments for Atopic Dermatitis: A Review of the Literature

**DOI:** 10.3390/jcm11174974

**Published:** 2022-08-24

**Authors:** Nikolaos Sideris, Eleni Paschou, Katerina Bakirtzi, Dimitra Kiritsi, Ilias Papadimitriou, Aikaterini Tsentemeidou, Elena Sotiriou, Efstratios Vakirlis

**Affiliations:** 1 First Department of Dermatology and Venereology, Aristotle University of Thessaloniki, 54643 Thessaloniki, Greece; 2 Department of Dermatology, Medical Center—University of Freiburg, Faculty of Medicine, University of Freiburg, 79104 Freiburg, Germany

**Keywords:** atopic dermatitis, topical treatment, JAK inhibitors, PDE-4 inhibitors

## Abstract

Atopic dermatitis (AD) is a chronic inflammatory dermatosis with periods of exacerbation and remissions. AD is characterized by intense, persistent pruritus and heterogeneity in clinical symptomatology and severity. Therapeutic goals include the amelioration of cutaneous eruptions, diminishing relapses and eventually the disease burden. To date, topical corticosteroids (TCS) and calcineurin inhibitors (TCI) have yet been deemed the mainstay of topical treatments in AD management. Nevertheless, despite their indisputable efficiency, TCS and TCI are not indicated for continuous long-term use given their safety profile. While research in AD has concentrated predominantly on systemic therapies, more than 30 novel topical compounds are under development. The existing data appear encouraging, with some regimens that are already FDA-approved (ruxolitinib was the most recent in September 2021) and several pharmaceutical pipeline products for mild-to-moderate AD that are in an advanced stage of development, such as tapinarof, difamilast and roflumilast. Larger, long-term studies are still required to evaluate the efficacy and safety of these novel compounds in the long run and weigh their advantages over present treatments. In this review, we aim to provide an overview of the latest knowledge about AD topical treatments, echoing upcoming research trends.

## 1. Introduction

Atopic dermatitis (AD) is a common inflammatory skin disease affecting as much as 25% of children and up to 10% of adults [1].

Prevalence depends mainly on genetic and socio-economic factors, with developed countries being more affected, while environmental factors, such as latitude and UV exposure, also play a role [2]. As the child grows, the disease improves or completely resolves in more than 50% of the patients over 6 years old. In some cases, nevertheless, AD persists or even starts in adulthood [3]. The main clinical characteristics of the disease are pruritus, eczematous lesions usually in age-specific body sites, dry skin and chronic courses with relapses and remissions.

AD has a multifactorial etiology, including immune system dysfunction, an impaired skin barrier and genetic and environmental contributing factors [4,5]. Although the interaction among those factors is not fully understood, it is clear that their synergy leads to a defective skin barrier that is unable to preserve moisture. Skin becomes dry, irritated, erythematous, exudative and prone to infection. Some lesions, after extensive scratching, become lichenified.

The goal of AD management is to treat the skin-barrier defect and inflammation and to restore the microbiome, thus obtaining prolonged patient remission. Topical therapies are key players in achieving those goals. They provide targeted anti-inflammatory activity and improve skin pathology with lower costs and increased safety compared to systemic treatments.

For more than 50 years, the cornerstone of topical AD treatment has been emollients and topical corticosteroids (TCS) [6]. In 2000, topical calcineurin inhibitors (TCI) were introduced, and no significant progress has been made ever since. Until recently, molecular targeting therapies began to emerge, and a revolutionary era started for medicine. Dermatology is one of the most privileged specialties in this field, given the plethora of biologics, Janus kinase and phosphodiesterase-4 inhibitors and other new molecules available for the treatment of various chronic diseases. In this review, we present the latest topical treatments for AD, including not only those that are already approved but also those in the pipeline. To find the data presented here, we conducted an extensive search in Medline, ScienceDirect and Google Scholar with various combinations of the search terms, including “Atopic Dermatitis”, “Eczema”, “treatment”, ”development”, “therapy” and “new”, ”emerging” and ”upcoming”. We also used the terms “JAK inhibitors”, “PDE-4 inhibitors”, “Aryl Hydrocarbon Receptor Agonists” and specific drug names found in the literature. The “Reference” section of relevant manuscripts was hand-searched to maximize the sensitivity of our search. We also searched the archives of major recent dermatology conferences and contacted some of the developers for information that we could not find elsewhere. Two authors (N.S. and E.P.) searched clinicaltrials.gov and clinicaltrialsregister.eu with Atopic Dermatitis as the only term. Several hundred studies were identified and searched one by one to find those concerning topical treatments. Apart from the data contained in the two registries, all identified drug names were used as search terms both in Medline and in websites for the general public/search engines to identify more medical literature and other information as press releases.

As this review includes many types of evidence, the risk of bias of included sources was not examined.

## 2. Janus Kinase Inhibitors

Janus kinases (JAKs) is a family of intracellular tyrosine kinases comprising four members (JAK1, 2, 3 and tyrosine kinase 2 [TYK2]). The JAKs, along with Signal Transducer and Activator of Transcription (STAT) proteins (STAT-1,-2,-3,-4,-5a,-5b and -6) and trans-membrane receptors are the three main parts of the JAK-STAT pathway [7].

A vast array of hormones, interferons, colony-stimulating factors and interleukins exert their actions through the JAK-STAT pathway [8]. Receptors for those factors rely on JAKs for downstream responses and subsequent modulation of gene expression. Pairs of JAKs, varying by receptor, bind to them with different results for each dimeric cytokine receptor–JAK pair. The now activated JAKs phosphorylate the receptors, forming a docking site for STATs. Those are then also phosphorylated and migrate to nucleus, affecting gene expression [9,10,11].

The vital role of JAKs in human physiology was evident upon their discovery. Involved in both hematopoiesis and immunity, JAKs became a treatment target for numerous diseases across various medical specialties [12,13].

The role of JAKs in the pathogenesis of AD is complex. They exaggerate Th2 cell response, activate eosinophils, suppress regulatory T cells (Tregs), upregulate epidermal chemokines, pro-inflammatory cytokines, etc. [14]. Building on this concept, their inhibition has been considered a promising treatment option.

### 2.1. Delgocitinib

On 23 January 2020, in Japan, delgocitinib became the first topical JAK inhibitor approved for the treatment of AD. Delgocitinib is a first-generation pan-JAK inhibitor blocking the activation of inflammatory cells (T and B), monocytes and mast cells and improving skin-barrier dysfunction [15]. Three major randomized phase-3 trials (RCTs) of topical delgocitinib for AD have been held: (i) JapicCTI-173554, (ii) JapicCTI-173555 and (iii) JapicCTI-184064 (www.clinicaltrials.jp, accessed on 1 June 2022).

In JapicCTI-173554, Japanese patients over 16 years of age with modified Eczema Area and Severity Index (mEASI) score > 10, Investigator’s Global Assessment (IGA) score of 3 or 4 and body surface area (BSA) involvement of 10% to 30% were eligible for enrollment. Part 1 was a 4-week, randomized, double-blind, vehicle-controlled study. After completion of part 1, patients could enter part 2, a 24-week, open-label extension study. Patients with worsening AD during part 1 could discontinue treatment or enter part 2 at the investigators’ discretion. In part 2, all patients received delgocitinib 0.5% ointment.

In part 1, the least-squares mean percent changes from baseline in mEASI score were −44.3% in the delgocitinib group and 1.7% in the vehicle group at the end of treatment. Reduction in mEASI score in the delgocitinib group started from week 1 and continued to week 4. IGA and pruritus Numerical Rating Scale (NRS) scores were also improved in the delgocitinib group compared with the vehicle group (*p* = 0.32 for the overall IGA score, 0.05 for the IGA face/neck score, 0.01 for NRS).

The proportion of patients with a mEASI-50 was 51.9% in the delgocitinib group and 11.5% in the vehicle group. The proportion of patients with a mEASI-75 was 26.4%in the delgocitinib group and 5.8% in the vehicle group.

Improvements in all AD parameters (mEASI, IGA, pruritus NRS scores and the percentage of patients with mEASI-50 and mEASI-75) persisted at the second part of the study. At week 24, the mean percent change from baseline in the mEASI score was −56.3%, and the proportions of patients with mEASI-50 and mEASI-75 were 69.3% and 35.8%, respectively [16].

JapicCTI −173,555 was an open-label, multicenter, phase 3 study, with Japanese patients over 16 years old with mild to severe AD (IGA 2 to 4, BSA 5–30%) and longer treatment durations (52 weeks). Improvement in all AD scores with delgocitinib 0.5% ointment b.i.d persisted for the study duration. The proportion of patients with mEASI-50 was 31.5% at week 4, 42.3% at week 24 and 51.9% at week 52. The proportion of patients with mEASI-75 was 10.9% at week 4, 22.7% at week 24 and 27.5% at week 52 [17].

The last study, JapicCTI-184064, evaluated the safety and efficacy of topical delgocitinib in pediatric patients aged 2 to 15 years old. Part 1 was a 4-week double-blind period in which patients were randomized in a 1:1 ratio to delgocitinib 0.25% ointment or vehicle. Part 2 was a 52-week open-label extension period. Eligible patients who received the vehicle treatment in part 1 were also treated with delgocitinib 0.25% or 0.5% ointment in part 2, according to the investigator’s judgment. In the first four weeks, 50.7% of patients in the delgocitinib group compared with 17.6% of patients in the vehicle group achieved a mEASI-50. mEASI-75 was achieved by 37.7% of patients in the delgocitinib group compared with 4.4% of patients in the vehicle group. In part 2, improvements in mEASI, IGA and pruritus scores persisted through week 56. Around 73.6% and 52.8% of the patients who received the drug in part 1, achieved mEASI-50 and mEASI-75, respectively. In patients who received vehicle in part 1, the percentage who achieved mEASI-50 and mEASI-75 at week 52 was 70.5% and 52.5%, respectively [18].

In all studies, adverse events (AEs) related to the drug were mostly mild. The most common AE was nasopharyngitis, followed by application site folliculitis and acne, influenza, Kaposi’s varicelliform eruption, herpes simplex, impetigo, fungal infections, molluscum contagiosum, etc.

Recently, a small, retrospective, 4-week Japanese study with 25 patients was published, in which delgocitinib was compared to topical corticosteroids as twice-weekly maintenance therapy in adults with AD [19].

AD parameters did not differ significantly between the two groups. Mean changes in NRS score and erythema index were slightly better in the TCS treated areas. However, the stratum corneum hydration in the delgocitinib group was maintained, while that of the TCS group worsened. The authors conclude that topical delgocitinib may be an effective maintenance therapy of AD in dry skin patients, sparing long-term corticosteroids usage.

### 2.2. Ruxolitinib

Ruxolitinib is a first-generation potent JAK1/2 inhibitor. Its oral form revolutionized the treatment of JAK2-driven myeloproliferative disorders and is currently FDA approved for polycythemia vera, myelofibrosis and steroid-refractory acute graft-versus-host disease [20].

The topical form was approved by the FDA on September 2021 for the short-term and non-continuous chronic treatment of mild to moderate AD in non-immunocompromised patients over 12 years of age. Topical Ruxolitinib Evaluation in Atopic Dermatitis (TRuE-AD) 1 and 2 were the two major, identical, phase 3 trials that confirmed the antipruritic and anti-inflammatory effects of the drug in patients with AD.

A total of 631 patients in TRuE-AD 1 and 618 in TRuE-AD 2 were randomized 2:2:1 to twice-daily 0.75% cream, 1.5% cream or vehicle cream for the first 8 weeks of the studies. Key inclusion criteria included age ≥ 12 years, diagnosis of AD for ≥2 years, IGA score 2–3 and BSA involvement of 3–20%. After the 8-week period, eligible patients continued treatment for an additional 44 weeks with 0.75% or 1.5% ruxolitinib cream. Patients initially randomized to the drug remained on their treatment and those randomized to vehicle were re-randomized to either cream regimen.

The primary endpoint of IGA 0–1 at week 8 was achieved by 50% and 39% of patients in TRuE-AD1 and TRuE-AD2, respectively, on 0.75% cream. For 1.5% cream, the percentage of patients achieving IGA 0–1 was 53.8% and 51.3%, while 15.1% and 7.6% (*p* < 0.0001) were achieved for vehicle only. EASI-75 at week 8 was achieved by 56.0% and 51.5% of patients on 0.75% cream, 62.1% and 61.8% on 1.5% cream and 24.6% and 14.4% on vehicle, respectively. Improvements in EASI-90 and NRS score were also significant (*p* < 0.05). It is worth mentioning that a reduction in itching started just 12 **h** after treatment initiation with 1.5% ruxolitinib cream [21].

The most common treatment-related AE was application-site burning sensation, mainly observed with vehicle (4.4%) than with 0.75% (0.6%) or 1.5% (0.8%) cream. None of the reported AEs were serious.

In a long-term safety period, up to week 52, patients were to treat skin areas with active AD only and stop treatment 3 days after clearance of lesions. They were to restart at the first sign of recurrence.

The proportion of patients with no/minimal AD lesions (IGA score 0/1) with ruxolitinib 0.75% and 1.5% cream ranged from 62.4% to 76.9% and from 66.5% to 77.3%, respectively, in TRuE-AD1 and from 59.6% to 76.7% and from 72.0% to 80.1% in TRuE-AD2 from weeks 12 to 52. In both studies, the mean measured total affected BSA was <3% throughout the long-term safety period in the ruxolitinib 1.5% cream and most of the period in the ruxolitinib 0.75% cream. Treatment-emergent AEs were reported in 60.1% and 53.8% patients who applied ruxolitinib 0.75% and 1.5%, respectively. Treatment-related AEs were reported in 20 patients (4.7%) who applied ruxolitinib 0.75% cream and in 13 patients (2.9%) who applied ruxolitinib 1.5% cream: none were serious, with upper respiratory tract infection, nasopharyngitis and influenza being the most common.

Treatment-emergent AEs resulted in discontinuations in nine patients (2.1%) in the ruxolitinib 0.75% cream group and no patients in the ruxolitinib 1.5% cream group. In summary, approximately 70% of patients maintained no or minimal lesions (IGA score 0/1) and the extent of AD lesions remained low during the 44-week extension period of TRuE-AD1 and 2. Ruxolitinib cream was well tolerated in the long-term setting, with no serious treatment-related AEs [22].

TRuE-AD3, a phase 3 randomized trial to assess the efficacy and safety of topical ruxolitinib in children aged 2 to 12 years old, is currently underway (NCT04921969).

### 2.3. Tofacitinib

Topical tofacitinib, a JAK 1/3 inhibitor, was evaluated in a 4-week, phase 2a, randomized study (NCT02001181). Sixty-nine adults with mild-to-moderate AD were randomized 1:1 to 2% tofacitinib or vehicle ointment b.i.d.

Percentage change from baseline (CFB) in EASI score at week 4 was the primary endpoint. Secondary endpoints included percentage CFB in body surface area (BSA), CFB in EASI Clinical Signs Severity Sum Score, the proportion of patients with Physician’s Global Assessment (PGA) response and CFB in patient-reported pruritus. Safety, local tolerability and pharmacokinetics were monitored.

The mean percentage change from baseline at week 4 in EASI score was −81.7% for tofacitinib and −29.9% for vehicle (*p* < 0.001). Similarly, all other efficacy endpoints were significantly improved with tofacitinib compared to vehicle (*p* < 0.001). Safety was comparable between regimens [23].

### 2.4. Brepocitinib

Brepocitinib, a JAK1/TYK2 inhibitor, was also evaluated in a phase 2 study (NCT03903822) with 240 adolescents and adults with mild-to-moderate AD. Patients were randomized to 6 weeks of treatment in one of eight study arms: once-daily topical brepocitinib at 0.1%, 0.3%, 1% or 3% concentration, twice-daily brepocitinib at 1% or 3% concentration or once or twice-daily vehicle cream.

The primary endpoint was a percentage change in EASI score from baseline to week 6. Brepocitinib 1% and 3% once daily and 1% twice daily achieved EASI score reductions of 70.1%, 67.9% and 75%, respectively, while the decrease was 44.4% and 47.6% among those in the once-daily and twice-daily vehicle control groups.

In the once-daily regimens, 29.7–44.4% of the patients achieved an IGA score of 0/1, compared to the 10.8% of the patients on once-daily and 13.9% on the twice-daily vehicle [24]. No phase 3 trial of brepocitinib in AD is currently active.

### 2.5. Other JAK Inhibitors

Another new agent is ATI-1777, a “soft” JAK1/JAK3 inhibitor. “Soft” JAK inhibitors are designed to provide JAK inhibition at the site of application and be rapidly metabolized in the systemic circulation. A phase 2 randomized study (NCT04598269) has been completed, but results have not been published yet. The developer has reported positive results in a press release [25], namely a 74.4% reduction in mEASI score from baseline at week 4 in the drug arm, compared to a 41.4% reduction in patients applying vehicle. No serious adverse events were reported

Ifidancitinib (ATI-502) is another JAK1/JAK3 inhibitor evaluated in a phase 2, open-label safety study (NCT03585296). Patients over 18 years old with moderate to severe AD and 2–20% BSA involvement were enrolled. They applied ATI-502 to affected areas b.i.d for 4 weeks. Seven out of twenty-two subjects reported 16 AEs, all unrelated to the drug. One person discontinued due to an unrelated bilateral lower extremity cellulitis, outside of the site of cream application.

Proportions of subjects with PGA of near clear with ≥2 grade improvement from baseline were 10.5%, 23.5% and 41.2% at weeks 1, 2 and 4. The percentage change from baseline in EASI was 18%, 35% and 40% at weeks 1, 2 and 4, respectively [26].

Studies for another two JAK inhibitors are currently active. NCT04435392 (phase 1/2) for jaktinib, a pan-JAK inhibitor, and NCT04717310 (phase 2/3) for ivarmacitinib (SHR 0302), a JAK1/STAT3 inhibitor. JAK inhibitors for the topical treatment of AD are summarized in Table 1.

## 3. Phosphodiesterase-4 Inhibitors

Over the last few years, PDE4 inhibitors have been identified as promising therapeutic agents for AD treatment [27,28]. The role of PDE4 activity in the circulating leukocytes in AD pathology is through the degradation of cyclic adenosine monophosphate (cAMP). The inhibition of PDE4 leads to increased levels of cAMP, which is involved in controlling the production of key inflammatory cytokines such as IL-4, IL-5, IL-10, IL-13 and prostaglandin E2 [29]. Moreover, the inhibition of PDE4 in monocytes in vitro enhances the cellular control of inflammation by downstream regulation of the nuclear factor-kB and nuclear factor of activated T-cell signaling pathways [30].

### 3.1. Crisaborole

Crisaborole 2% ointment, the first topical PDE4 inhibitor, was licensed by the FDA in December 2016 for the treatment of mild-to-moderate AD in children 2 years and older and in March 2020 for infants 3 months of age and older. Two 28-day, randomized, double-blind, vehicle-controlled trials (AD-301: NCT02118766, AD-302: NCT02118792) assessed the efficacy and safety for patients aged 2 years or older with mild-to-moderate AD. The primary endpoint was clear (0) or almost clear: (1) Investigator Static Global Assessment score (ISGA) with a greater than or equal to 2 grade improvement on day 29. Twice-daily applications led to a 32.8% (AD-301) and 31.4% (AD-302) reduction in ISGA compared to 25.4% and 18% for vehicle-treated subjects (*p* = 0.038, *p* < 0.001), respectively [31]. Moreover, in a phase 4 open-label study of 137 infants 3 to less than 24 months with mild-to-moderate AD, 30.2% of the patients achieved the primary endpoint of the ISGA with a safety profile equal to that in older children [32].

More common AEs related to crisaborole 2% ointment were local pain, burning, and stinging [31,32]. The mechanism that causes pain is still unknown. In a real-world retrospective study, more patients reported pain than in phase 3 clinical trials (31.7% versus 4.4%). Fifty percent of them applied crisaborole exclusively to the face (*p* = 0.048) [33]. An open-label 48-week safety extension study demonstrated that crisaborole had a favorable long-term safety profile without increasing the risk for treatment-related adverse events [34]. In the same study, 77.8% of patients did not require rescue therapy, defined as the need for the concomitant, nonconcurrent use of low-to mid-potency TCS or TCIs. Other important points regarding the treatment of AD with crisaborole include the rapid improvement in pruritus (as early as 24 h after the first application) and the normalization of epidermal pathology toward nonlesional skin [35].

### 3.2. Difamilast (OPA-15406)

PDE4 inhibitor OPA-15406 is a novel topical treatment for AD with high selectivity for phosphodiesterase-4-B. The effectiveness and tolerability were evaluated in a phase 2 study. Patients who met eligibility criteria were randomized into three groups to receive OPA-15406 0.3%, OPA-15406 1% or vehicle. OPA-15406 1% cream achieved the best results (IGA 0 or 1) at week 4 at a rate of 20.9% versus 14.6% for 0.3% cream and 2.7% for vehicle [36]. Recently, results from a phase 3 study have been published [37]. Patients aged 15 to 70 years received difamilast 1% ointment or vehicle twice daily for 4 weeks. The primary endpoint of IGA 0–1 with ≥2-grade improvement at week 4 was achieved by 38.46% of patients in the ointment group, compared to 12.64% in the vehicle. No serious adverse events were reported.

### 3.3. E6005 (RVT-501)

For the PDE4 inhibitor E6005, also known as RVT-501 or lotamilast, results are available from phase 1/2 trials in Japan. In early phase 1 trials in healthy adults and patients with AD, this compound was well tolerated with minimal systemic exposure [38]. In phase 2 dose-finding studies, 40 adult male patients randomized to receive E6005 ointment (0.01%, 0.03%, 0.1% or 0.2%). The targeted lesion severity scores significant decreased in the 0.2% group [39]. Subsequently, in a vehicle-controlled trial, 78 adult patients were randomized to receive either the 0.2% E6005 ointment or vehicle. The reduction in EASI and SCORAD scores from baseline after 12 weeks was significant in the E6005 group [40].

### 3.4. Other PDE-4 Inhibitors

In a randomized, vehicle-controlled, phase 2a trial, topical roflumilast (ARQ-151) 0.5% demonstrated no significant improvements in EASI score; however, there was a significant decrease in pruritus as measured by the NRS score (NCT01856764) [41,42]. Currently, roflumilast 0.15% cream is trialed (NCT04156191) for children and adults with AD. Furthermore, roflumilast 0.3% has shown very promising results in trials for psoriasis. Phase 2 studies have been completed for other topical PDE4 inhibitors, DRM02, Hemay808 and PF-07038124, but results have not been published to date. Phase 1 studies have been completed for LEO-39652 and LEO-32731, also known as orismilast, but results are not available yet. PDE-4 inhibitors for the topical treatment of AD are summarized in Table 2.

## 4. Aryl Hydrocarbon Receptor Agonists

The aryl hydrocarbon receptor (AhR) is a member of the Pern-Arnt-Sim (PAS) superfamily of transcription factors. They detect and respond sense diverse endogenous and exogenous molecules and impact multiple biological activities [43]. Initially, AhR was recognized as the mediator of the toxic effects of dioxins, but consequently, other ligands were identified. In response to activation by a ligand, AhR translocates from the cytoplasm to the nucleus, where it controls the transcription of a wide variety of target genes [44].

AhR is broadly expressed in the skin, and when activated, it upregulates the gene expression of filaggrin, loricrin and involucrin, accelerating epidermal terminal differentiation [45]. Consequently, AhR plays a vital role in developing and maintaining the skin barrier, epidermal homeostasis, pigmentation and the response to external signals, such as UVB, phytochemicals, environmental toxins or microbial products [46,47].

Although the mechanism of action was unknown, humans used AhR agonists empirically for thousands of years in numerous dermatologic conditions, the most classic example being coal tar [48]. Today, targeting the AhR system is an up-and-coming field for developing new drugs in dermatology and in many other specialties. Recently (23 Μay 2022), tapinarof was approved by the FDA for the treatment of plaque psoriasis in adults [49].

### Tapinarof

Tapinarof is a naturally derived small molecule produced by bacterial symbionts (Photorhabdus luminescens) of entomopathogenic nematodes of the genus Heterorhabditis. It regulates skin-barrier protein expression, has antioxidant activity and suppresses IL-17 and -22 [47].

A randomized, multicenter, phase 2b, double-blind, vehicle-controlled study (NCT02564055) is the most important one that has been completed for AD. In this study, 247 adults and adolescent patients (12 to 65 years old, BSA between 5% and 35%, and IGA score ≥ 3) were randomized to receive tapinarof cream (0.5% or 1%) or a vehicle control, either once daily or twice daily for 12 weeks with a 4-week follow-up period.

At week 12, IGA response rates (IGA 0 or 1) were 53% (1% twice daily), 46% (1% once daily), 37% (0.5% twice daily) and 34% (0.5% once daily) versus 24% (vehicle twice daily) and 28% (vehicle once daily). This improvement was maintained for the 4-week follow-up period. Overall, among patients treated with tapinarof cream, 1% showed higher response rates than the 0.5% groups. EASI75 was also significantly higher in the tapinarof-treated groups at week 12: 60% (1% twice daily), 51% (1% once daily), 51% (0.5% twice daily) and 39% (0.5% once daily) versus 26% (vehicle twice daily) and 25% (vehicle once daily). The same was also true for EASI90 at week 12: 43% (1% twice daily), 27% (1% once daily), 28% (0.5% twice daily), and 22% (0.5% once daily) versus 14% (vehicle twice daily) and 5% (vehicle once daily). BSA, pruritus NRS, subject impressions of AD and Patient-Oriented Eczema Measure (POEM) scores were also significantly improved in tapinarof groups [50].

Treatment-emergent AEs (TEAEs) were reported in 51% of patients, and the majority were mild to moderate in intensity. The most frequently reported TEAE was nasopharyngitis. The other TEAEs reported in at least 5% of patients in any arm or in total were folliculitis, worsening or flare of AD, upper respiratory tract infection, headache, acne and impetigo. Overall, 32 patients (13%) had TEAEs that were considered treatment-related.

More patients in the vehicle groups (6 of 82 [7%]) than in the groups treated with tapinarof (7 of 165 [4%]) discontinued the study because of TEAEs. Worsening or flare of AD was the most frequent TEAE that led to a discontinuation of the study treatment. ECG findings were observed in 21% of patients in the groups treated with tapinarof and 17% in the vehicle groups. They were not considered significant, resolved over time and never led to treatment discontinuation. Elevations in liver enzyme levels (at least twice the upper limit of the normal range) were observed in six patients treated with tapinarof. They all resolved during treatment, and none of those patients discontinued treatment [51].

Three large (estimated enrollment: 961, 400 and 400 patients) phase 3 studies (NCT05142774, NCT05014568 and NCT05032859) are currently active.

It is worth mentioning here a common misconception about tapinarof synonyms. At the beginning of its development, the drug was known as GSK-2894512, WBI-1001 and DMVT-505. A common error by many authors is that benvitimod is considered the same as tapinarof. Indeed, the active ingredient is the same (3,5-dihydroxy-4- isopropyl-trans-stilbene) and is isolated from the bacterium Photorhabdus luminescens [52]. However, tapinarof cream 1% comprises a novel vehicle with specific excipients to enhance efficacy, drug delivery and patient acceptability. Benvitimod 1%, uses a petrolatum-based vehicle for delivery and requires twice-daily dosing. Clinical trials for the two formulations are also separate with essential differences. Benvitimod is currently trialed in China for AD (NCT05326672) and is approved for psoriasis [53].

## 5. Transient Receptor Potential Vanilloid 1 Antagonists

The transient receptor potential channels (TRPs) are a superfamily of ion channels consisting of six members (TRPC, TRPV, TRPP, TRPM, TRPA and TRPML). TRP proteins are remarkable channels because of the diversity of their activation mechanisms, cation selectivity and biological function [54].

Member 1 of the TRPV family (TRPV1) is a nonselective cation channel with high permeability to calcium. It is activated by heat, low pH, capsaicin and endogenous inflammatory mediators [55]. It is expressed, among others, in keratinocytes, mast cells and cutaneous sensory nerves, indicating its important role in cutaneous physiology and disease [56]. TRPV1 has shown to play a role in pruritus, epidermal barrier function and inflammation [57]. In AD lesions, TRPV1 is overexpressed and its activation results in the production of molecules that promote itch and inflammation [58].

### Asivatrep

Since preclinical experiments in rats and mice, asivatrep (then known as PAC-14028) demonstrated promising results, positively affecting many aspects of AD. Its use suppressed serum IgE, mast cell degranulation, the expression of IL-4 and IL-13, itch and inflammatory cell infiltration. Skin-barrier recovery was accelerated, and possible carcinogenicity was ruled out [55,59,60,61].

Later, a phase 2b study (NCT02757729) was conducted on 194 adults (19–70 years old) with mild to moderate AD. Patients were randomized in asivatrep 0.1%, 0.3%, 1.0% or vehicle, twice daily for 8 weeks. The primary efficacy variable was the IGA success rate (IGA score of 0 or 1 with at least a two-grade improvement from baseline) at week 8. Secondary efficacy variables included SCORAD and EASI scores, pruritus VAS and sleep disturbance scores.

The IGA success rates were 14.6% for vehicle cream, 42.6% for 0.1% cream, 38.3% for 0.3% cream and 57.5% for 1.0% cream. All secondary variables were also improved, although the improvement was not statistically significant. The small sample and adult age of patients may have been important factors affecting statistical insignificance. The safety profile and the overall incidence of AEs were similar in the drug and vehicle groups [62].

Recently, results of a phase 3 study were published [57]. In it (CAPTAIN-AD and NCT02965118), 240 patients 12 to 70 years old were randomized 2:1 to 1.0% asivatrep or vehicle cream for 8 weeks.

At week 8, the proportion of patients with an IGA score of 0 or 1 was 36.0% in the asivatrep group and 12.8% in the vehicle group (*p* < 0.001). Improvements of at least 2 points on the IGA from baseline score was 20.3% versus 7.7% (*p* = 0.01). At week 8, the reduction in EASI score was 44.3% versus 21.4%, respectively (*p* < 0.001). Additionally, significantly more patients in the asivatrep group experienced an improvement of at least 50% (EASI-50), 75% (EASI-75) and 90% (EASI-90) (50.3%, 23.5% and 9.8% versus 28.2%, 11.5% and 2.6%). Pruritus and sleep disturbance were significantly reduced in the drug group compared to the vehicle (*p* = 0.02).

Asivatrep cream was well-tolerated and was not associated with clinically significant application site reactions. Overall, the incidence of TEAEs was reported in 14.7% of patients treated with asivatrep and 6.3% treated with vehicle cream. The most common TEAEs were nasopharyngitis (2.6%), urticaria (1.3%), burning sensation (1.3%) and rhinorrhea (1.3%), which were similar in the vehicle group. No patient discontinued treatment due to adverse events, and serious adverse events were not reported.

Overall, in clinical trials, asivatrep cream resulted in evident and enduring positive results in treating AD, along with an acceptable safety profile.

## 6. Skin Microbiome Modulators

In the 1970s, it was shown that Staphylococcus aureus (S.aureus) is overrepresented on the skin of AD patients [63]. Later, cutaneous dysbiosis was identified as a contributing factor to AD pathogenesis [64]. Cutaneous dysbiosis is characterized by an increased colonization of S.aureus and reduced colonization by the abundant bacterial genera of healthy skin.

More recently, patients colonized with S.aureus have been described as a unique AD phenotype. Patients in this category have more severe disease, reduced barrier function, increased allergen sensitization and elevated IgE, eosinophils, lactate dehydrogenase and various Th2 biomarkers such as TARC, periostin and CCL26 [65,66].

S.aureus toxins induce mast cell degranulation, promoting innate and adaptive immune responses, and induce IL-1b production from monocytes. In the dermis, through the defective skin barrier, S.aureus interacts with immune cells and triggers cytokine production including IL-4, IL-13, IL-22 and TSLP [67].

Those observations led to various strategies to try and modulate the skin microbiome of AD patients, either by decreasing S.aureus or increasing normal microbiota [68].

In healthy individuals, Roseomonas mucosa (R.mucosa) is the most representative Gram-negative bacteria [69]. An open-label trial (NCT03018275) with topical application of R.mucosa twice-weekly for 6 weeks in both adults and children (9–14 years old) with AD found that the commensal bacterium was associated with improvements in SCORAD and pruritus and a reduction in TCS use, with no significant AEs [70]. In the same trial, it was noted that non-responders had a family history of AD persisting into adulthood for at least 3 generations, suggesting that heritable factors may influence responses to R. mucosa therapy.

Another topical formulation, FB-401 with three strains of R.mucosa, showed promising results in a phase 1/2 trial. Sixty percent of adult patients showed a 50% reduction in SCORAD, while 90% of the pediatric patients achieved EASI50 and 30% achieved EASI90 [68]. Unfortunately, in a later, more extensive trial, FB-401 failed to meet the primary goal of EASI50 (58% in the FB-401 arm versus 60% in the placebo arm), and development will not continue [71].

Staphylococcus hominis A9 (ShA9) is another healthy human skin microbiome bacterium that has been trialed as a topical therapy for AD. ShA9 killed S.aureus on the skin of mice and inhibited the expression of the toxin psmα that promotes inflammation. Then, in a phase 1, randomized, 1-week trial (NCT03151148), topical ShA9 or vehicle was applied on the forearm skin of 54 adults with S. aureus-positive AD. The primary endpoint of safety was met, and a small improvement of AD lesions was also induced, rendering ShA9 a safe and potentially beneficial future treatment [72].

Nitrosomonas eutropha is an ammonia-oxidizing Gram-negative bacterium able to produce nitric oxide, which is an important mediator with beneficial metabolic and potential anti-inflammatory properties [67]. Results of three phase 1 and 2 trials (NCT04490109, NCT03775434 and NCT03235024) are not yet available, although pruritus and AD appearance were significantly improved in adults and children, according to a press release [73].

One of the reasons AD patients have a predisposition for cutaneous and systemic infections is the decreased antimicrobial peptides production [74]. Omiganan is a synthetic indolicidin analogue. Indolicidin is an antimicrobial peptide isolated from the neutrophils of cows. It is active against Gram-positive and -negative bacteria but has also been shown to kill fungi and even HIV [75]. Cationic peptides, such as omiganan, are also suggested to have immunomodulatory roles in both pro- and anti-inflammatory pathways.

Because of those properties, omiganan gel is investigated as a possible treatment for various infectious and inflammatory disorders, among them some cutaneous ones, including acne, rosacea, condylomata acuminata and vulvar intraepithelial neoplasia (VIN) [74].

For AD, a phase II trial randomized 36 patients with mild to moderate disease 1:1:1 to omiganan gel 1%, 2.5% and vehicle, once daily for 4 weeks. Small but significant results in BSA, SCORAD and pruritus were observed only in the 2.5% arm. Skin microbiota shifted from lesional to non-lesional [76]. In a later trial by the same group, omiganan gel 2.5% twice daily led to a recovery of dysbiosis but without clinical improvement [77]. The authors concluded that dysbiosis does not seem a viable monotherapy drug target in mild-to-moderate AD.

Omiganan development in the future may focus on diseases where S.aureus plays a more central role, e.g., in superinfected AD, reducing the need for oral antibiotics, or eradicating multi-drug resistant S. aureus strains in long-term carriers.

The niclosamide ATx201 can also achieve the decolonization of S. aureus. In a phase 2 trial (NCT03304470), 31 patients with mild-to-severe AD received ATx201 cream 2% and a matching vehicle once daily for 3 weeks. Treatment was generally safe and the histological and transcriptional profiling analysis on day 22 demonstrated that treatment significantly increased the expression of biomarkers related to the skin-barrier function and decreased expression levels of markers related to inflammation [78].

Primary and major secondary endpoints of the most important trials for the above-mentioned therapeutic targets are summarized in Table 3.

## 7. Newer Emolients

Emollients and moisturizers are the cornerstone of basic disease management of AD [79,80]. Fourteen independent publications from Europe, North America, Asia, the Asia-Pacific region and Australia, including AD management guidelines between 2007 and 2022, were reviewed and displayed that daily moisturization is an integral part of recommendations [81,82].

These products contain vehicle-type substances such as humectants (urea or glycerol) and occludents (petrolatum) and act as an occlusive layer on the skin, promoting stratum corneum hydration and reducing transepidermal water loss [79,83,84]. Data from Cochrane review with emollient trials display a favorable impact on AD management with no superiority among them [85].

In recent years, there has been a rapid increase in non-medicated emollients containing active ingredients termed “emollient plus” or AD therapeutic moisturizers that improve skin barrier with antipruritic, anti-inflammatory and antioxidant effects. Active ingredients are, for example, ceramides, saponins, colloidal oatmeal and nonpathogenic bacterial lysates from Aquaphilus dolomiae or Vitreoscilla filiformis with possible molecular targets as it emerges from in vitro and clinical research data [86,87,88]. A prospective, double-blind, placebo-controlled clinical study demonstrated that patients with mild AD who received cream with 5% Vitreoscilla filiformis decreased SCORAD levels and pruritus significantly compared with those who received placebo [89]. These lysates might influence the skin microbiome due to the reduction in Staphylococcus aureus colonization and display immunomodulatory effects locally on the skin. Thus, they could be a therapeutic approach targeting prevention of relapses and stabilization of AD skin [90].

## 8. Other

Many other molecules are under investigation for the topical treatment of AD. Those with trials that have not started yet are ongoing, or those with results that are not yet available are presented in Table 4.

## 9. Discussion

AD is the most common inflammatory skin disease with a considerable impact on the lives of patients and their families. It imposes a substantial physical, psychological and social burden. Pruritus and the accompanying sleep disturbance are distressing and increase the risk for psychiatric conditions such as ADHD, depression, suicidal ideation and autism. Multiple other complications and comorbidities have been reported, including, but not limited to, growth delay, bacterial and viral infections, ocular abnormalities, aortic stiffness and other allergic, metabolic and autoimmune conditions [1,2]

Nevertheless, for decades and until recently, the only options for the topical treatment of the disease were TCS and TCIs in combination with emollients. Although effective, significant concerns about their long-term usage bother clinicians and patients. TCS may cause skin atrophy, telangiectasia and numerous other adverse effects. Systemic absorption is also a concern, especially in younger children with a greater surface-to-weight ratio than adults. TCIs are less effective than TCS in controlling exacerbations of the disease. Additionally, the FDA black box about the theoretical increased risk of malignancy distressed many parents of children with AD. The multifaceted and, to an extent, still unknown pathophysiology; inconsistent clinical manifestations depending on age, body sites affected and other factors; and the chronic course with relapses and remissions added to the difficulty of managing the disease.

This unpleasant situation is rapidly changing for the better. The pathophysiologies of AD, inflammatory pathways and physiology, in general, are better understood due to advances in basic science research. At the same time, research fields regarding drug development are also advancing swiftly. AD or dermatology, as well as medicine in general, is experiencing a revolution in therapeutics, which began with biologics. Since then, progress seems to have accelerated, and the future is eagerly awaited. New drug categories that we mentioned in this review and many others in other specialties are or will be available in the times ahead.

Subsequently, other important steps are advancements in safety, more effective production and, therefore, lower cost, discovering biomarkers and an improved understanding of the various phenotypes and/or behaviors of the disease in different patients. Ultimately, clinicians will not only have many treatment options but also the knowledge to use them in a non-blind manner by targeting specific aspects of the disease in each patient. The result will be improved, including the individualized treatment of AD and other chronic, complex diseases.

Finally, concerning AD, another urgent necessity in the coming years is the comparison of efficacy and safety between all new and upcoming treatments. Although TCS and TCIs were the only available topical treatments for decades, they are indeed effective and inexpensive. The adverse events mentioned above are not frequent if properly used. Therefore, it will be difficult for many of the upcoming treatments to outplace the old ones until their efficacy, safety, tolerability, adherence and cost-effectiveness are proven by comparison studies [91]. Until now, in most studies of new treatments, the comparator is usually the vehicle. Since the industry can generally obtain approval for new drugs without head-to-head studies, real-life data comparing new and old topical treatments will be valuable in the following years. Until then, a level of confusion and uninformed choices by clinicians and patients is expected. Network meta-analyses can, to a certain degree, fill this knowledge gap. For example, a recent meta-analysis from China comparing JAK and PDE-4 inhibitors concluded that tofacitinib 2% b.i.d, ruxolitinib 1.5% b.i.d and delgocitinib 3% b.i.d showed superior efficacy over other JAK and PDE4 inhibitors [92]. Future real-life data will also specify another important aspect of the new treatments, namely if any of them is better at targeting specific phenotypes of the disease. In most clinical trials and those presented here, patients are recruited just by having the required conditions for the trial severity of the disease. More information, such as IgE levels, filaggrin mutation status, other atopic comorbidities, etc., are usually unmentioned. Regardless, progress in the topical treatment of AD is of major importance since even the safest systemic agent has significantly more safety concerns than topical agents, especially in children.

In summary, in this review, we aimed to make a substantial amount of information about an exciting and fast-growing field of dermatology easily accessible to anyone interested, including emerging topical therapies for AD. We focused on the most important information, major therapeutic targets and principal aspects of trial results, namely safety and efficacy. It is evident that the landscape will change dramatically soon enough, albeit many important questions remain to be answered. The upcoming approval of the presented topical therapies (and many more systemic ones) will result in a wide range of available treatment options and will enable personalized decision making depending on patient characteristics, making clinicians’ and patients’ lives easier.

It is still impossible to predict the role that each one of all the presented agents will have in the treatment of AD in future. It seems unlikely that only one agent will replace TCS in being the gold standard for the majority of patients. Responses in trials are varying, and the heterogeneity of the disease is now better understood. This knowledge and all other aspects of the disease, such as age, age of onset, comorbidities, etc., together with new discoveries in the stratification of AD cases (biomarkers and artificial intelligence) will lead to the best possible treatment for each patient.

## Figures and Tables

**Table 1:** JAK inhibitors for the topical treatment of AD.

Name	Selectivity	Phase	Age/Severity	Regimens
Delgocitinib	pan-JAK	approved (Japan)	approved for children 2–15, adults >16, moderate-to-severe	0.25% children 0.5% adults, b.i.d
Ruxolitinib	JAK1, 2	approved (USA)	approved for >12, on trial for 2–12, mild-to-moderate	1.5% b.i.d
Tofacitinib	JAK1, 3	II	18–60, mild-to-moderate	2% b.i.d for 4 weeks
Brepocitinib	JAK1, TYK2	II	12–75, mild-to-moderate	0.1%, 0.3%, 1%, 3% q.d, 0.3%, 1% b.i.d for 6 weeks
ATI-1777	JAK1, 3	II	18–65, moderate-to-severe	2% b.i.d for 4 weeks
Ifidancitinib	JAK1, 3	II	>18, moderate-to-severe	0.46% b.i.d for 4 weeks
Jaktinib	pan-JAK	II	18–65, mild-to-moderate	0.5%, 1.5%, 2.5% b.i.d, 2.5% q.d
Ivarmacitinib	JAK1	III	>12, mild-to-moderate	0.5%, 1%, 2% b.i.d

**Table 2:** PDE-4 inhibitors for the topical treatment of AD.

Name	Phase	Age/Severity	Regimens
Crisaborole	approved	>3 months, mild-to-moderate	2% b.i.d
Difamilast (OPA-15406)	III	15–70, mild-to-moderate	1% b.i.d for 4 weeks
Lotamilast (E6005, RVT-501)	II	20–64, all	0.2% b.i.d for 12 weeks
Roflumilast	II	18–65, moderate. 3 months—17 years currently on trial	0.5% b.i.d for 15 days, 0.05% and 0.15% for 4 weeks currently on trial
DRM02	II	18–70	0.25% b.i.d for 6 weeks
Hemay808	II	18–65, mild-to-moderate	1%, 3%, 7% for 29 days
PF-07038124	II	18–70, mild-to-moderate	0.01% q.d for 6 weeks
LEO-39652	I	>18, mild-to-moderate	3 weeks
Orismilast (LEO-32731)	I	>18, mild-to-moderate	3 weeks

**Table 3:** Primary and major secondary endpoints of the most important trials for the drugs mentioned in Section 2, Section 3, Section 4, Section 5 and Section 6.

Drug	Primary End-Point	Other End-Points
Delgocitinib	JapicCTI-173554: Mean percent change in mEASI at week 4: −44.3% in the drug group vs. 1.7% for vehicle. (*p* < 0.001). JapicCTI-173555: Safety: AEs in 69% of patients. 15.4% considered treatment-related. 1.4% considered serious (Kaposi’s varicelliform eruption)	JapicCTI-173554: mEASI-50 at week 4: 51.9% for drug vs. 11.5% for vehicle (*p* < 0.001). mEASI-75 at week 4: 26.4% vs. 5.8% respectively (*p* < 0.01). IGA response rates at week 4: *p* = 0.32 for overall score, *p* < 0.05 for face/neck score. NRS: lower in drug group. All results maintained at week 24. JapicCTI-173555: mEASI-50 at week 4, 24, 52: 31.5%, 42.3% and 51.9%. mEASI-75 at week 4, 24, 52: 10.9%, 22.7% and 27.5% IGA and NRS: improved at weeks 4, 24 and 52
Ruxolitinib	IGA 0–1 at week 8: 53.8%(TRuE-AD1) and 51.3% (TRuE-AD2) in the 1.5% cream groups vs. 15.1% and 7.6% for vehicle (*p* < 0.0001)	EASI-75 at week 8: 62.1% and 61.8% in the 1.5% cream groups vs. 24.6% and 14.4% for vehicle (*p* < 0.0001). EASI90 at week 8: (*p* < 0.0001) vs. vehicle. Reduction in NRS: (*p* < 0.05) vs. vehicle
Tofacitinib	EASI score change at week 4: 81.7% vs. 29.9% for vehicle.	EASI 50, 75 and 90: Significantly higher for drug vs. vehicle (*p* < 0.05) at weeks 2 and 4. Change in BSA: −76% for drug vs. −31% for vehicle, significantly greater (*p* < 0.001) at week 4. ISI scores: significantly greater for drug vs. vehicle at weeks 2 and 4 (*p* < 0.001).
Brepocitinib	EASI score change at week 6: 70.1%, 67.9%, and 75%, for the 1%, 3% q.d and 1% b.i.d groups respectively. 44.4% and 47.6% in the q.d and b.i.d vehicle groups.	IGA score of 0/1 at week 6: 27.8–44.4% of patients on q.d drug vs. 10.8% for q.d vehicle. EASI 90 at week 6: 27.8–41.7% of patients on 0.3%, 1%, and 3% q.d cream, vs. 10.8% for q.d vehicle, 27% of patients on 1% b.i.d cream, vs. 8.3% b.i.d vehicle. Improvement of at least 4 points on the PP-NRS at week 6: 45.2% of patients on 1% cream q.d, 50% on 3% q.d, and 40.7% on 1% b.i.d, vs. 17% for vehicle.
ATI-1777	Reduction in mEASI score at week 4: 74.4% in the drug arm, vs. 41.4% for vehicle	not yet available
Ifidancitinib	PGA of near clear with ≥2 grade improvement: 10.5%, 23.5%, 41.2% of patients at weeks 1, 2, and 4.	Change in EASI: 18%, 35%, 40% at weeks 1, 2, and 4. Percent change in SPA: 35%, 46% and 31% at weeks 1, 2, and 4.
Jaktinib	PGA 0/1 or a decrease of ≥2, 7 days after the last dose: not yet available	PGA 0/1 at 8 and 16 weeks: not yet available
Ivarmacitinib	Change in EASI at Week 8: not yet available	not yet available
Crisaborole	ISGA score 0/1 with ≥2 grade improvement at day 29: 32.8% (AD-301) and 31.4% (AD-302) reduction vs. 25.4% (*p* = 0.038) and 18% (*p* < 0.001) for vehicle.	ISGA score 0/1 at day 29: 51.7% vs. 40.6% (*p* < 0.005) and 48.5% vs. 29.7% (*p* < 0.001) respectively. Time to ISGA success: 14.7% for drug vs. 5.4% for vehicle at day 8. Median time to improvement in pruritus: 4 days for drug vs. 9 days for vehicle. Mean change in DLQI at day 29: −5.2 for drug vs. −3.5 for vehicle.
Difamilast (OPA-15406)	IGA 0–1 with ≥2 grade improvement at week 4: 38.46% of patients in the ointment group vs. 12.64% for vehicle (*p* < 0.0001)	EASI 50, 75, 90 at week 4: 58.24%, 42.86% and 24.73 of patients in drug group vs. 25.82%, 13.19% and 5.49% for vehicle. Mean percent change in EASI score at week 1: −32.6% vs. −10.4% for drug and vehicle respectively (*p* < 0.0001). POEM, affected BSA, pruritus VRS, Skindex-16: all significantly improved vs. vehicle (*p* < 0.0001) at week 4
Lotamilast (E6005, RVT-501)	Long-term safety and tolerance: Neither death nor serious TEAEs were encountered in the entire study period. In the randomization phase, the incidence of TEAEs was 50.0% in the drug group vs. 38.5% for vehicle group. The incidence of TEAEs leading to study withdrawal was 9.6% in the drug group and 15.4% for vehicle group.	Scores reduction at week 12: significantly reduced: EASI, *p* = 0.030; SCORAD-objective, *p* < 0.001; SCORAD-C, *p* = 0.038) Not significantly reduced: Itch Behavioral Rating Scale, (*p* = 0.462)
Roflumilast	Change in Modified Local SCORAD at day 15: Not significant reduction vs. vehicle (*p* = 0.276)	Change in PAP at day 15: Significantly reduced (*p* < 0.013)
DRM02	not yet available	not yet available
Hemay808	not yet available	not yet available
PF-07038124	not yet available	not yet available
LEO-39652	not yet available	not yet available
Orismilast (LEO-32731)	not yet available	not yet available
Tapinarof	IGA response rates at week 12: 53% (1% b.i.d; *p* = 0.008), 46% (1% q.d; *p* = 0.084), 37% (0.5% b.i.d; *p* = 0.240), and 34% (0.5% q.d; *p* = 0.535) vs. 24% (vehicle b.i.d) and 28% (vehicle q.d).	EASI75 at week 12: significantly higher in the tapinarof groups, except the 0.5% q.d, vs. vehicle groups. EASI90 at week 12: significantly higher in the tapinarof groups, except the 0.5% b.i.d, vs. vehicle groups. Mean percent change in EASI at week 12: significantly higher in all tapinarof groups vs. vehicle groups. Mean percent change in BSA at week 12: significantly greater in the tapinarof groups, except the 0.5% b.i.d, vs. vehicle groups.
Asivatrep	IGA score of 0 or 1 at week 8: 36.0% in the drug group vs. 12.8% for vehicle.	Improvement ≥2 points on IGA score at week 8: 20.3% for drug vs. 7.7% for vehicle. EASI reduction at week 8: 44.3% vs. 21.4% respectively. EASI-50, 75, and 90 at week 8: 50.3%, 23.5%, and 9.8% of patients on drug vs. 28.2%, 11.5%, and 2.6% on vehicle. Statistical significance achieved in all secondary end-points, as also in pruritus and sleep disturbance reduction.
R.mucosa	50% improvement in SCORAD: 66.7% of patients	75% improvement in SCORAD: 40% of patients. Subjective pruritus: significantly decreased.
FB-401	EASI50: 58% in drug arm vs. 60% in placebo arm	-
ShA9	Safety through day 8 compared to vehicle: Significantly fewer AEs in participants treated with ShA9 (*p* = 0.044)	EASI and SCORAD: no significant difference Decrease in S. aureus and increased ShA9 DNA: endpoints met
Nitrosomonas eutropha	Not yet available, positive results in pruritus and AD appearance reported in press release	-
Omiganan gel	*S.aureus* reduction at day 28: Statistically significant in the omiganan 1% (*p* = 0.03) and 2.5% (*p* = 0.01) vs. vehicle.	Clinical improvement evaluated by EASI, SCORAD, IGA, POEM, DLQI and NRS: no improvement
ATx201	Safety: safe and well tolerated	Expression of biomarkers related to skin-barrier function: Significantly increased (*p* < 0.05). Histological responders: 51.7% of those receiving 2% cream vs. 31.0% for vehicle.

ISI: Itch Severity Item, PP-NRS: Peak Pruritus Numerical Rating Scale, SPA: Subject’s Pruritus Assessment, DLQI: Dermatology Life Quality Index, VRS: verbal rating scale, PAP: Participants’ Assessment of Pruritus.

**Table 4:** Agents for the topical treatment of AD for which trials have not started yet, are ongoing or results are not available in ClinicalTrials.gov or clinicaltrialsregister.eu.

Agent	Mechanicm of Action	NCT ID
ALX-101 Gel 1.5% (Rovazolac)	LXR agonists	NCT03175354
AM1030-CREAM	5-HT2BR antagonist	NCT02379910
AMTX-100 CF	Nuclear transport modifier (NTM)	NCT04313400
ASN008 (*1)	Targets small afferent sodium channels/Antipruritic	NCT03798561
Atuzabrutinib (SAR 444727 or PRN 473)	BTK inhibitor	NCT04992546
Aurstat Hydrogel	Emolient/Antipruritic	NCT01905631
BEN2293	TRK inhibitor	NCT04737304
BioLexa	Antibacterial	NCT04544943
BMX-010	Antioxidant	NCT03381625
BPR 277	Kallikrein-related peptidase	NCT01428297
BX005-A (*2)	Phage cocktail targeting S.aureus	NCT05240300
CD 5024/Ivermectin (Soolantra)	Chloride channel agonists	NCT03250624
CYCLATOP(Cyclosporine 5% solution) (*3)	Calcineurin inhibitor	NCT02865356
DBI-001 (*4)	Antibacterial	NCT05253755
DMT210 Topical Gel	G protein-coupled receptor agonist	NCT02949960
DS 107/DGLA (*5)	Bioactive lipid (dihomo-γ-linolenic acid) inhibiting the expression of CD40	NCT02925793 NCT03676036 NCT03676933
Ectoin Dermatitis Cream 7% (EHK02)	Emolient	NCT04097327
FMX114 (tofacitinib and fingolimod) (*6)	Jak inhibitor and sphingosine-1-phosphate receptor modulator	NCT04927572
GM-XANTHO	Βotanical drug balm	NCT04369846
HAT01	Botanical complex	NCT03089229
HL-009 Liposomal Gel(Cobamamide)	Vitamin B12 analogues, Nitric oxide inhibitor.	NCT01568489
HY209 Gel/Taurodeoxycholic acid	G Protein Coupled Receptor 19(GPCR19) agonist	NCT04530643
IDP-124	Undefined mechanism	NCT03058783 NCT03002571
Isopentenyltheophylline 0.44% + Glycerin 4.56%	Undefined mechanism	NCT05057351
Jaungo (Shiunko in Chinese) (*7)	Herbal ointment	NCT02900131
Lactibiane Topic AD	Εmolient/Cosmetic product	NCT04728269
Lactobacillus reuteri (ADreuteri)	Probiotic	NCT04265716
Levagen+/ Palmitoylethanolamide (PEA)	Endocannabinoid-like lipid mediator	NCT05003453
Menthoxypropanediol	Anti-TRPM8/Antipruritic	NCT03610386
MH004	Unknown	NCT04815148
NLAC (Natural Lactic Acid-enriched Cream)	Emolient	NCT05092464
PR022 (Hypochlorous acid)	Antiseptic	NCT03351777
Q301(Zileuton) (*8)	leukotriene inhibitor	NCT03571620 NCT02426359
RelizemaTM cream	Antioxidant/ Antipruritus	NCT05259774
SB414 (Berdazimer sodium) (*9)	Nitric oxide donors	NCT03431610
SB011	GATA3 transcription factor inhibitor	NCT02079688
SNG100	Unknown	NCT04615962
TER-101	Unknown	NCT04753034
Topialyse Baume Barrière (TOPIA)	Emolient	NCT05006300
ZEP-3Na	synthetic analogue of a compound of rattle snake venom	NCT04307862
ZK245186 (Mapracorat)	Selective glucocorticoid receptor agonists (SEGRAs).	NCT01228513 NCT00944632 NCT01359787
0.5% Cannabidiol and 1% Hemp Oil (Celosia)	Emolient	NCT04045314
2.5% and 5% Cis-urocanic Acid	Emolient	NCT01320579

LXR: liver X receptor, 5-HT2BR: serotonin receptor 2B, TRPM8: Transient receptor potential cation channel subfamily M(melastatin) member 8, HAT01: herbal anti-inflammatory treatment; S.aureus: Staphylococcus aureus; TRK: tropomyosin receptor kinases; BTK: Bruton’s tyrosine kinase. Results from various sources (press releases, conference posters or the literature) are available online for agents marked with * in Table 4, as follows (All accessed on 17 June 2022): *1 https://www.asanabiosciences.com/_files/ugd/d170b0_f8d4c69d2e374ce99f41e2d734cb78dc.pdf; *2 https://www.biomx.com/wp-content/uploads/2022/01/a11y-RAD-2021-Poster_June-2021F.pdf; *3 https://www.mdedge.com/pediatrics/article/175673/atopic-dermatitis/topical-cyclosporine-safely-tamed-atopic-dermatitis-4; *4 https://www.dermbiont.com/in-the-news/2021/1/8/dermbiont-announces-positive-results-in-phase-2a-clinical-trial-in-atopic-dermatitis-with-a-topical-live-biotherapeutic; *5 https://www.dsbiopharma.com/2018/10/03/ds-biopharma-announces-positive-top-line-phase-2b-trial-results-for-ds107-as-a-topical-treatment-for-mild-to-moderate-atopic-dermatitis/; *6 https://vynetherapeutics.com/pipeline-overview/fmx114/; *7 https://pubmed.ncbi.nlm.nih.gov/30219454/; *8 http://www.qurient.com/bbs/content.php?co_id=q301; *9 https://novan.com/novan-to-present-data-from-sb414-phase-1b-atopic-dermatitis-clinical-trial-at-3rd-inflammatory-skin-disease-summit/.
